# The de-perimeterisation of information security: The Jericho Forum, zero trust, and narrativity

**DOI:** 10.1177/03063127231221107

**Published:** 2023-12-28

**Authors:** Matt Spencer, Daniele Pizio

**Affiliations:** 1 University of Warwick, Coventry, UK

**Keywords:** information security, semantics, narrativity, trust, sensemaking

## Abstract

This article analyses the transformation of information security induced by the Jericho Forum, a group of security professionals who argued for a new ‘de-perimeterised’ security model. Having focused on defensive perimeters around networks, early 2000s information security faced a growing set of pressures: the maintainability of firewalls given increasing traffic volume and variety, the vulnerability of interior network domains, and the need to cope with and enable new working arrangements and ways of doing business. De-perimeterisation was a radical rethinking of the nature of security and created the conditions for the rise of ‘Zero Trust’ architectures. This shift has radical implications for the architectures of digital infrastructures that undergird many aspects of contemporary life, the risks to which people and societies are exposed, and the nature of work and business in a digital economy. We develop a semiotic analysis of the Jericho Forum’s interventions. Using insights from material semiotics, security theory and the theory of narrativity, we argue that de-perimeterisation can be understood as a shift in security logic, or, a shift in how security can (be made to) make sense. We examine a cluster of images used by the Jericho Forum, and analyse how they challenged the coherence of perimeter-based thinking and provided the materials for constructing a new model. We argue that a focus on the narrative dimension of security provides a window into fundamental semantic transformations, reciprocal historical relations between semantics and technical change, the *agencement* of security technologies, and determinations of value (what is worth securing).

‘De-perimeterisation’ refers to a recent and radical transformation in information security, with deep implications for the design of digital infrastructures, the kinds of risk imposed on societies, how business is conducted, and the nature of work in digital environments. Yet the concept is almost unheard of beyond the field of information security. This article addresses this gap, presenting an analysis of the interventions of the Jericho Forum, a temporary organization that existed between 2003 and 2013, set up to act as the herald for this new way of thinking and doing security. The Jericho Forum was founded by a group of senior security leaders at large corporations, with a shared mission of campaigning for the de-perimeterisation of information security, a process that would involve a transformation in how information technology was secured and how security practitioners understood their work.

At its heart, de-perimeterisation involves a shift away from the perimeter-based model of security that had become established in the 1990s, in which the role of information security was understood in terms of protecting the boundary of an organization’s private network, keeping threats away from this ‘home territory’. By the early 2000s, the Jericho Forum would argue, this view was outdated: Connectivity was ubiquitous, traffic volumes and complexity were growing, organizational arrangements were increasingly hybrid, working patterns fluid, and infrastructures needed to be constructed in agile fashion from hybrid public/private architectures. A new model of security was needed for this de-perimeterised world.

We develop our analysis as a study in the semantics of security, an examination of the historical mutation in what security means. We attend to processes of sensemaking (Weick et al., 2005) and to interventions that disrupt stable categories. To do this, we bring together insights from material semiotics, security studies, and the Greimasian theory of narrativity. We examine how a set of key metaphors or images deployed by the Jericho Forum served to perturb established logics of the perimeter and to create new possibilities that would go on to shape digital infrastructures today. These are images of: (1) the city of Jericho’s prophesied falling walls, (2) a market for security technology in need of a catalyst, (3) sieve-like porous boundaries, (4) an opportunistic global enterprise, exploiting internet connectivity to reach new markets, and (5) ink-stained cash from a booby-trapped cash canister (and which itself served as inspiration for the very concept of de-perimeterisation and raised provocative questions about what it is that ought to be secured).

We begin with a discussion of securing and sensemaking. This sets up our conceptual apparatus and the theoretical justification for our approach. The empirical part of our paper begins with background on the origins of the perimeter, and moves to an examination of the Jericho Forum and the five images. We comment on how de-perimeterisation took hold and close with a discussion of the implications for security and trust in contemporary digital society, and our understanding of the relationships between technical and conceptual change. We aim to show that processes of narrativization, through which securing is (re)organized as a meaningful activity, are just as fundamental in the evolution of information security as are processes of technical change.

## Securing, semantics, and sense

Interpretive processes, especially those involved in giving account of particular configurations of technology, people, and processes *as secure*, play a vital role in determining whether and how such configurations hang together. This ‘hanging together’ that makes up infrastructures and other macro actors (Callon & Latour, 1981) is thus an effect requiring both functional efficacy (i.e. how functional components inter-operate) and the efficacy of sensemaking, that is, the ability to ‘tell a story’ about a technical architecture, business process, design decision, etc., that accounts for it as secure.

Accounting for a system as secure has become a ubiquitous passage point in system development, taking place in various ways, such as reporting to regulators and corporate boards, in technical design reviews, release approval boards, investor meetings, incident post-mortems, and various kinds of security audits. In STS, the concept of ‘accountability’ has ethnomethodological roots (where it refers to the sense immanent to practices) and has been adapted to apply to practices of ‘giving account’, and where the coherence of the account is explicitly in question (Woolgar & Neyland, 2013). It is through such practices that architectural configurations gain normative legitimacy and are thus justified for implementation, ongoing maintenance, integration into other structures, or being turned toward new uses. Thus, even in a field so reputedly technical as information security, the coherence of technical architectures is produced *narratively*.

Assembling a coherent narrative in which some system is presented as secure means drawing on what security theorist Balzacq (2005) calls the ‘semantic repertoire’ of security: those images, anecdotes and principles available in that time and place for story construction. Such a repertoire, from the material semiotic point of view, does not primarily exist ‘in the head’, but must be seen as a real distribution of material signs duplicated, iterated, adjusted, adapted, and circulated. Some refer directly to technologies in use, while others create metaphorical associations across domains. Balzacq’s interest in semantic repertoires arises from a discussion of the legacy of the ‘Copenhagen School’ of security studies, which in the 1990s gave centre stage to the discursive construction of security problems and solutions, and emphasized the performativity of security (Buzan et al., 1998). The ability to effectively declare something a ‘security situation’ is treated as a pivotal political act, serving to justify and make possible interventions like military action or exceptional policing arrangements. The study of these political speech acts also led to a recognition of the importance of historically contingent ‘logics’ of security, schematic ways in which security makes sense.

Security sensemaking depends on the ability to assemble tropes from a semantic repertoire in the service of a security logic, and logics may emerge, change over time, conflict and compete. Doty’s (1998) analysis of 1990s US immigration debates, for instance, shows logics of national security, societal security, and human security being brought into tension with each other. For Foucauldian thinkers, such logics are imagined in a neo-Kantian light as setting a historical ‘grid of intelligibility’ in which security problems can be formulated, and without which they would be unthinkable as such (Collier et al., 2004; Collier & Lakoff, 2015, p. 26). Foucault’s (2007) account of security as an apparatus discriminates between sovereign logics based on control over territory and biopolitical logics for governing a population. Collier & Lakoff extend this account, tracing the emergence of a new ‘vital systems security’ through the 20^th^ century and moving from military doctrines of strategic bombing, systems theory, and nuclear preparedness, to health systems security, disaster response, and critical infrastructure protection. This develops and deploys a particular image of society as comprised of inter-linked systems vulnerable at critical junctures (Collier & Lakoff, 2015)﻿. What count as security problems and solutions are thus produced historically, in processes that can span many domains of thought and practice.

For the Jericho Forum in the early 2000s, reliance on the perimeter was not only an increasingly ineffective approach to information security; it was also an obstacle to the emergence of any alternative paradigm. The perimeter had become a default trope, overdetermining the meaning of security. To de-perimeterise is thus to challenge the perimeter’s *black boxing* capabilities: the way it holds together a security logic and particular kinds of security architectures, all as a largely unexamined default. A black box ‘contains that which no longer needs to be reconsidered, those things whose contents have become a matter of indifference’ (Callon & Latour, 1981, p. 285). To de-perimeterise is thus to find points of difference that reopen the question of what securing means in this domain. Compared with securitization and Foucauldian approaches, we narrow in on a semiotic microcosm, a particular juncture of contestation. We treat the Jericho Forum as semiotic *bricoleurs*, collaging a set of images (textual and visual) drawn from various areas of life (history, religion, and even cooking) and capable of de-perimeterising because of their ability to challenge unexamined ideas, disrupt the coherence of existing schemas, and furnish a repertoire for a new logic.

We draw on the analytical resources of the Greimasian theory of narrativity to develop our argument and go beyond furnishing a simple inventory of old and new semantic repertoires (1987). A security logic can be understood as what Greimas called a ‘narrative schema’, a relational configuration of actants: the object that needs protection, the anti-subject that is a threat, the ‘modal objects’ that serve as the means of securing, the ‘sender’ that is the source of the necessity of securing, the ‘subject’ doing the securing, and so on. (Cooren, 2000, ch.3; Greimas, 1987; see also Baldwin, 1997; Smith, 2005). Thinking through narrativity helps us examine how security logics ‘hang together’ and how they change. We refer here to *agencement* (Muniesa et al., 2007), a process in which an entity comes to have agent-like characteristics, and which corresponds to the kind of schematic translation where an entity takes the role of subject. It is precisely such a process that provokes the Jericho Forum into action: Perimeter technologies such as firewalls, having become ubiquitous, start to look less like a mere tool and more like the ‘doer’ or subject of securing.

The Greimasian approach also directs our attention to the figurative level of analysis, where we can unpack the stakes of the metaphorical baggage that an entity brings along. Hence the perimeter also comes to determine the nature of the object of protection through its own spatial logic, so that what needs to be protected, the object of value, is the domain that the perimeter encloses. De-perimeterisation, therefore, must ‘open the black box’ of the metaphor, challenge these spatial logics of enclosure and reposition the subject who secures. In doing this, de-perimeterisation raises anew the question of what it is that information security protects, a question to which the Jericho Forum responds by fostering a new and deeper alignment between information security and asset value.

Adopting this analytical approach also allows us to take advantage of synergies between material semiotics and narrative or communication-centred approaches to organization (Taylor & van Every, 1999). This much is already anticipated by our use of the term ‘sensemaking’ to describe processes of assembling semantic coherence. ‘To focus on sensemaking is to portray organizing as the experience of being thrown into an ongoing, unknowable, unpredictable streaming of experience in search of answers to the question, “what’s the story?”’ (Weick et al., 2005, p. 410). Sensemaking can thus be understood as organizing things into accountable, narratable order. It is in this tradition of organization studies that we find the most developed readings of Greimasian narrativity as a theory of significant action, the story-like structure of endeavours. For Cooren (2000, p. 60), narrativity is ‘co-constitutive with a project’, meaning by this term both an organized activity and a projection towards a future resolution. ‘If our actions can be *articulated* and *coordinated* in a series of events that seem to overwhelm us,’ writes Cooren, ‘it is because we agree to *insert* our actions in different *narrative schemas* that *a priori* structure our interactions’ (p. 3). The study of security logics and how they are contested, then, is the study of the (re)organization of security.

A number of scholars have preceded us in foregrounding the role of narrative in studies of security. These include the concept of ‘narrative power’ developed in international relations (Hagström & Gustafsson, 2019), in which political situations are examined for the interplay between minor stories, ‘counter-narratives’, and hegemonic ‘grand narratives’. Narrative analysis has been a powerful tool for examining how events are framed from different standpoints, and how the normal and exceptional are thus constituted in discourse (Wibben, 2011). In earlier work, one of us drew on narrative analysis to examine mistrust of security evaluations as enacted by sceptical stories told by information security practitioners (Spencer, 2022a). Here we develop this further, examining the role of narrativity in the fundamental process of constructing and deconstructing security.

There are also important precursors in wider STS engagements with information security in terms of its characteristic images and metaphors (e.g. Helmreich, 2000). Scholars adopting the standpoint of sociotechnical imaginaries also ask similar questions about the constellations of ideas and practices in which securities make sense—such as Tidwell and Smith’s (2015) analysis of US energy security. In recent years, scholars drawing on research in STS have examined the constitution of meanings of security through micro-analyses of security practices (Ermoshina & Musiani, 2018; Monsees, 2020), while in discursive analysis, scholars have looked at the interplay of disputed definitions of security in multi-stakeholder governance (Wolff, 2016). STS scholarship has moreover provided critical contributions to examining when and how dissonances emerge between security interventions and security logics used to justify them. Suchman et al. (2017) develop this line of critique in relation to the military use of drones and automated data-driven tracking and targeting: Such an apparatus is based on a pivotal but ultimately incoherent categorical differentiation of persons into ‘civilian’ or ‘combatant’ categories. Slayton (2021) has articulated a similar kind of critique, arguing that insecurities are created due to ‘contradictions between conceptions and practices of governance’ in information security (pp. 85–86). Our goal here is to examine how such dissonance emerges in an endemic fashion, driving historical differentials and internal reflexive dynamics that shape the field of information security from within (Spencer, 2021).

## Methodological note

This article is based on extensive documentary analysis of the archives of the Jericho Forum, as well as interviews with four founding members of the group, and analysis of materials they made available to us. We conducted an open-ended qualitative analysis of these materials, looking to identify the most important elements of the semantic repertoire. Of interest were those images that perform the work of characterization (characterizing the Jericho Forum as an agent), that challenge the intuitive coherence of perimeterised security, and that furnish the field with elements of an alternative, de-perimeterised security logic. We identified five such images used by the Jericho Forum, some of which perform more than one of these tasks. The tale of the city of Jericho’s falling walls, for instance, challenges the perimeter, but also serves to cast the Jericho Forum as prophets. The image of ink-stained cash challenges the simple notion of defending an interior, and provokes explication of how destruction may nevertheless preserve value.

We present this work as an analysis of a historical transformation. Some degree of complication is introduced by the fact that we are analysing an intervention in sensemaking that depends fundamentally on its own reflexive historiography, on the ability of the Jericho Forum to establish a historical differential between ‘before’ and ‘after’, between the old world of the perimeter and the de-perimeterised future. While we follow this narrative, broadly arguing in line with the Jericho Forum that there has been a shift in security logics, we also indicate points of potential discrepancy along the way. For instance, the advocates of the perimeter were not quite so naïve as they were later made out to be. There were also projects that ran in parallel to the Jericho Forum, such as the US military ‘Black Core’ architecture project, that were also influential in creating the conditions for these changes. Furthermore, it is important to recognize that we are examining an Anglophone discourse, and one that draws on Judeo-Christian cultural tropes. A different story can be expected to play out in other locales, in other political, social, and cultural contexts. More work is also needed in connecting these shifts in information security with contemporaneous changes in security domains such as the policing of territorial borders. We offer our analysis of the Jericho Forum, then, in anticipation of a wider discussion, focused on unpacking these relations.

## Before de-perimeterisation

### Crunchy shells and chewy centres

Scholars looking back at the early internet note that it ‘was designed in simpler times, when the user community was smaller, it was reasonable to trust most users and it was possible to trace and deal with misbehaviour’ (Clark et al., 2005, p. 93). That situation changed in the late 1980s and 1990s, with growing volume and variety of users, the arrival of business, and increasing awareness of malicious activities (DeNardis, 2007, p. 686).

The computing environments operated by private companies had likewise undergone dramatic transformations. The 1980s had seen the dominant architectural model shift from multi-user mainframe computing to networked computer environments. By the late 1980s, many organizations had accumulated large distributed systems in some cases made up of tens of thousands of machines, with the diversity of operating systems and protocols bringing substantial challenges of interoperability (DeNardis, 2007, p. 683; 2014, p. 68; Edwards, 1998, p. 22). Connecting these electronic ‘walled gardens’ to the internet brought further challenges and raised new questions about security.

Early incidents had raised awareness that interconnectivity brought risks, due to the possibility of attacking a remote system through hacking techniques and rudimentary malware: In 1983, a group of high school students known as the *Milwaukee 414s* illegally accessed US Department of Defense networks, while in November 1988, the ‘Morris’ worm caused a slowdown of the entire internet (Warner, 2012). These episodes ‘clearly signalled the end of an open and benign Internet’ (Ingham & Forrest, 2002, p. 4), indicating the online presence of ‘many untrusted and even malicious users’ (p. 6), a far cry from the small academic community whose practices, based on principles of self-management and decision-making autonomy, had governed the development of Arpanet since 1969 (Carlini, 2002).

The security of internet-connected private networks emerged as a specific concern as news of these episodes spread, carving out a distinct area within the broader computer security problem-space. Access control had been recognized in the 1960s and 70s as the definitive security challenge raised by multi-user computing environments (MacKenzie & Pottinger, 1997). Cryptography was another established security domain, developed around the problem of ensuring the confidentiality and integrity of sensitive communications, that had been revolutionized by computing. Against this background, the distinctive problem of securing interconnected networks emerged with its own emblematic technology: the firewall.

A firewall is a boundary device used to establish a security perimeter around a network of computers managed by an organization (called a Local Area Network or LAN), so as to isolate it from possible external threats located in computer networks outside its control (generically referred to as a Wide Area Network or WAN). The influential ‘Computers at Risk’ report commissioned by the Defense Advanced Research Projects Agency (DARPA) in the late 1980s saw the strategy as very general. ‘The principle of “divide and conquer” suggests that it may be wiser to divide a large system into smaller parts and to restrict severely the ways in which these parts can interact with each other’, for instance, by allowing ‘only certain limited kinds of traffic (i.e. email, not ftp)’, forbidding ‘fully general communication across the perimeter’ and specifying ‘the pairs of source and destination systems that can communicate through it’ (National Research Council, 1990, p. 266). Such devices make complexity manageable by allowing a conceptual separation of parts from the whole, creating ‘interior’ network regions connected to, but protected from, the outside.

The term ‘firewall’ dates back to the 18th century and was coined ‘to describe walls which separated the parts of a building most likely to have fire (e.g. a kitchen) from the rest of the structure’, thus preventing or slowing down the spread of a fire (Ingham & Forrest, 2002, p. 2). However, before this metaphor of precautionary architecture took hold, an image of a confection had already been attached to the boundary apparatus, one that would serve over the years as a reminder of persistent doubts about the efficacy of the approach. In his account of the exemplary ‘gateway’ machine he had set up at AT&T to control access from their private network to the ARPANET, Bill Cheswick (1990) referred to the approach as creating ‘a sort of crunchy shell around a soft, chewy centre’ (p. 2). The security of the interior relies on the efficacy of the perimeter, both as an infrastructure and as a way of thinking that assumes the hostile actors are outside, rather than already operating within.

By the time the Internet Architecture Board (IAB) met for their 1994 workshop on security, the general moniker of ‘firewall architectures’ had become conventional. However, the memo from the IAB meeting notes that such architectures had been ‘a very emotional topic’, and explicitly relates this to the concept of the ‘chewy centre’, the concern that firewalls ‘foster a false sense of security, leading to lax security within the firewall perimeter’ (Braden et al., 1994, p. 8). Whereas encryption and access control had both stimulated major efforts to make systems provably secure, the debate around firewalls was always contested, and had a much more pragmatic edge: a matter of prioritizing where constrained security resources should be invested.

The 1994 IAB report notes that firewalls had become a fact of life for organizations, and attributes this widespread adoption to the practicality of work. ‘In some sense,’ the authors write, ‘firewalls are not so much a solution to a security problem as they are a reaction to a more basic software engineering/administration problem: configuring a large number of host systems for good security’ (Braden et al., 1994, p. 10). The concentration of security work in the perimeter can thus be understood as, in part, a response to the complexities associated with the rise of heterogeneous distributed computing, a challenge of configuration that would go on to drive the development of automation in other domains (Spencer, 2022b). The concentration of security work in the perimeter did nevertheless create a significant workload for the growing profession of IT security, tasked with ensuring that firewall configuration and software was kept up to date and aligned with the needs of the organization (Cheswick & Bellovin, 1994, p. 52).

The firewall of a building and the firewall of a computer system use logics of containment in seemingly opposite ways: the former contains the fire within the limited area of the kitchen, while the latter protects the LAN by keeping unregulated traffic out. What they have in common is the use of spatial relations to protect value. Instead of wrapping protection around specific objects of value (in the LAN) or objects of risk (in the kitchen), the firewall separates and locks down encompassing spatial domains. In the digital space organized by the firewall, the relationships between objects are thus structured by an idea that associates the concept of movement with that of danger: Their function is to block the spread of a fire inside a building or the unauthorized diffusion of information from a computer system. The logic of the perimeter, then, is a logic of space and movement: spaces separated via control of movement, and the objects and persons they contain are bundled together as similarly valued, similarly trustworthy or similarly risky.

## De-perimeterisation

### Image 1: The falling walls of Jericho

By the year 2000, a fresh wave of sceptical discourse was starting to emerge, reviving earlier critiques and articulating novel concerns about the adequacy of the perimeter for current and future circumstances. Particularly vocal was a group of senior security leaders based largely in the UK, who had been among the first information security professionals to have been brought up to the top tier of the corporate hierarchy, as ‘CISOs’ (Chief Information Security Officers). Having met on a regular basis for some time to share knowledge and engage with technology developers and vendors, their collective concerns about the dominance of a perimeter-based mindset led in 2003 to the formalization of an explicitly anti-perimeter interest group, the Jericho Forum.

For the next 10 years, the members of the Jericho Forum produced documents, principles, blueprints and whitepapers, delivered outreach presentations at conferences, sponsored a ‘Jericho Challenge’ design competition, hosted events, and made extensive efforts to engage with the business media, writing for websites and giving interviews with journalists, all with the stated intention of capturing ‘the imagination of many of our industry’s decision-makers and influencers’ (Jericho Forum, 2007a). Their goal was to disassemble the perimeter, to reveal it to be inadequate to the challenges of the emerging digital economy, the challenges of open networks, mobile workforces, expanded outsourcing, dynamic and global business relationships, quickly and cheaply established commercial presence, greater connectivity, and the risks of ‘insider threat’ and social engineering attacks. The Jericho Forum were observers, oracles, and agents of de-perimeterisation. To them the perimeter was not just a technology; it had become a harmful mindset that needed to be supplanted. What they wanted was nothing less than the articulation of a new discourse, a new framing of what securing ought to be, and for information security *a de-perimeterisation of the mind*.

The name they took evokes an image with hidden complexity. It is a reference to the biblical tale of the Israelites’ assault on the ancient city of Jericho: Following God’s instructions to march around the city and for seven priests to blow trumpets on the seventh day, the Israelite army witnessed the city’s high walls miraculously crumble, opening the way for the destruction of the city. The image, drawing on one of the more brutal parts of the Old Testament, is hardly subtle in its evocation of the traditional security motif of physical protection, of security as a fortified boundary defending an interior. But exactly how is the biblical tale supposed to map onto early 2000s information security? Were the Jericho Forum members in the position of the Israelite army or in that of the embattled city? On the one hand, the Jericho Forum cast themselves as Old Testament prophets, publishing in 2007 the *Jericho Forum Commandments*, a set of 11 normative declarations addressed to the information security profession, the latter thus characterized as subjects of transcendent Law. Together with their foretelling of falling walls, this places the Jericho Forum clearly on the side of the Israelites. On the other hand, they, and the profession as a whole, also occupy the position of the people of Jericho, facing an enemy that many erroneously believed could be stopped by walls: Like the inhabitants of the city, they urgently needed to wean themselves off this dependency and mindset if impending doom were to be averted.

Like the core metaphor, the idea of de-perimeterisation had two faces, and this led to some communication challenges. The group’s members were agents of de-perimeterisations, pushing the agenda and challenging perimeter-thinking through many channels. But they were at pains to emphasize that de-perimeterisation was already a reality, an existing situation to which all needed to respond. Paul Dorey, CISO of Barclays Bank and later of British Petroleum (BP) and one of the founding leaders, reflects:

I had people [who] came out to me and said ‘So, how do you intend to de-perimeterise?’ No, it is not something you decide to do, or don’t. You are doing it. And you will do increasingly. … It came in [from the] strategic planning side of things … If this curve continues, this level of adoption, this level of business model continues the way we are expecting to, then … in 3 years’ time I am going to be struggling with a control model which is actually broken. (Paul Dorey, interview, 2022)

The same dual character was evident in the core text of the group, the ‘commandments’ published in 2007, between the ‘de-perimeterised future’ they heralded, and the ‘de-perimeterised vision’ they aimed to create. ‘The Jericho Forum (2007b) commandments define both the areas and the principles that must be observed when planning for a de-perimeterized future. Whilst building on “good security”, the commandments specifically address those areas of security that are necessary to deliver a de-perimeterized vision’ (p. 1). The document concludes with an enigmatic reflection on this complex implied temporality: ‘De-perimeterization has happened, is happening, and is inevitable’ (Jericho Forum, 2007b, p. 2). The Jericho Forum thus presented itself as pursuing two interlinked projects: conveying to the community that the perimeter had become obsolete (de-perimeterisation as a historical condition), and articulating a new security model (de-perimeterisation as a transformation of security thinking).

### Image 2: A catalyst for the market

When the Jericho Forum was established, the group represented itself as a ‘catalyst to accelerate the achievement of the Vision’—that is, ‘to enable business confidence for collaboration and commerce beyond the constraint of the corporate, government, academic and home office perimeter’—‘by defining the problem space, communicating the collective Vision, challenging constraints and creating an environment for innovation, demonstrating the market, influencing future products and standards’ (Bleech, 2005, p. 22). These latter points were crucial: The group argued forcefully that perimeter thinking was blocking product and standard developers from designing the kinds of technologies that were really needed.

If many of the technologies that would support a de-perimeterised security architecture did not yet exist, this was not because they were figments of glossy-eyed futuristic imaginings: To the Jericho Forum’s members, the problem was an obstruction in sensemaking. Perimeter-based thinking was accused of skewing the research and development efforts of product vendors. As Paul Simmonds, CISO of Motorola, Imperial Chemical Industries (ICI) and AstraZeneca (AZ) between 1995 and 2010, and Jericho Forum founder, put it at the close of a 2004 presentation: ‘[H]ow sure are we that the security/computing industry will deliver if we do not tell them what we want?’ (Simmonds, 2004, p. 18).

Many of the technologies required were already available in some basic form, but faced deployment challenges associated with scaling and interoperability. Among those explicitly sought were systems for better control over network topologies, including network virtualization, partitioning, and the compartmentalization of subnets. Comprehensive infrastructure was needed for identity management, including identity policy management, so that fine-grained control over user/service activities could be achieved. Similarly, it would be necessary to have anomaly detection systems capable of monitoring and facilitating response at a holistic network level. Perhaps surprisingly, firewalls were also on the list, but with an emphasis on device-level firewalls rather than network gateways, and to be accompanied by better device-level encryption and monitoring. Bringing all this together were a series of challenges of compatibility, language, and standards. The security policies configured into these infrastructures needed a common language that could be shared across organizations, so that they could seamlessly integrate where needed, and could be shared across technology vendors to ensure interoperability and avoid vendor-specific specialization in the profession. Standards for processes of user authentication, vital for managing networks of heterogeneous levels of risk, would be needed for similar reasons, as would general alignment of inter-organizational assurance processes to aid collaboration, communication and integration of processes and systems across organizational boundaries.

This projection of market need, with its portrayal of a problematically under-delivering market, was central to how the Jericho Forum presented its own agency, its ability to intervene in a global problem space by ‘catalysing’ the market. Vendors and product developers were poised to invest in and exploit new market niches, if only these opportunities could be made visible to them. Furthermore, the fact that the founding members were leaders at large global corporations (Royal Mail, ICI, BP and Standard Chartered Bank) placed four kinds of capital at their disposal. First, they had faced some of the most complex information security problems, so they could claim access to a sort of prescient ‘future present’ knowledge of what was needed. Second, they were embedded in contexts with significant resources available for scrutinizing the adequacy of solutions. Third, they were speaking on behalf of global corporations and could rely on their brand power and reputation to attract the attention of a wide audience of IT professionals. Fourth, and perhaps most important, they wielded huge buying power, and spoke as frustrated customers with chequebooks on the table.

‘What companies such as Microsoft are planning years from now’ claimed David Lacey (founder of the Jericho Forum, former principal technology consultant at Shell and director of security and risk management at Royal Mail), ‘we want it in two years’ time, or even today … [W]e need to speed it up, otherwise the solutions will appear after they are needed’ (Thomas, 2004). Simmonds framed the problem to us in slightly different words:

If you want in 3 years time [to be] doing something different, you need to influence what is in the R&D labs today. … We said ‘Let’s form a discussion group, think tank, talking sharp pressure group, to actually raise this up to a level and actually if [what] they see on the table is millions of dollars of spend … We took those commandments, the principles, whatever you wanted to call them, you go out to the vendors with them and say them ‘here is what we are trying to solve, here are the principles: Now build against that and then you will satisfy all of us. And look, here is our budget. Thirty global organizations with whopping great budgets: If you build it, we will buy it’. (Paul Simmonds, interview, 2022)

### Image 3: Your border is actually a sieve

Perimeters are secure to the extent that they control comings and goings: In this respect, doors and gates are as essential to the edifice as the solid structure. One of the primary arguments against the perimeter as a focus of information security was that it had become so porous that it no longer provided comprehensive control. ‘Your border is actually a sieve, keeping out the lumps—keeping out the script-kiddies’, as Simmonds put it in a 2004 interview (Saita, 2004). The border had not lost all value, but it provided only part of what was needed, and the community needed to grasp it as sieve-like rather than wall-like. ‘Our borders are ineffective today. We consider them more as sieves—they keep the lumps out, the script kiddies and denial-of-service attacks, but they’re not protecting us against many of the threats we face today’ (Simmonds, quoted in Cummings, 2004).

This image of a porous boundary was deployed in part to communicate the experience of managing network security for large scale organizations, where the Jericho Forum members had faced increasing data volumes, growing numbers and sophistication of network protocols, more complex routing through firewalls, and increasing use of end-to-end encryption, all limiting the possibilities of control at the perimeter. Maintaining firewall configurations became increasingly difficult. Simmonds reflects on the experiences in the 1990s that informed his push towards de-perimeterisation.

[In the 1990s,] AstraZeneca had 137,000 active IP addresses inside the corporate perimeter. What rules did I have in my firewall? And the answer: ‘Well, I have got two and a half thousand [and] I did not know what they are. And they are not documented! (Paul Simmonds, interview, 2022)

The image of porous boundaries was also associated with organizational changes, the growing need to interconnect and interoperate with partner organizations. For those from the oil and gas sector, for instance, it was necessary to integrate data flows from companies carrying out seismic surveys or maintaining oil rigs, and to have employees working from other companies’ premises. As CISOs, says Dorey, ‘we had pressure of people inside the corporation needing to access systems outside, and people outside the corporation network needing to access systems that were being run by the corporation.’ Eventually, ‘the perimeter started to become a little bit grey because at some point you are not using systems that are nicely delineated’ (Paul Dorey, interview, 2022).

Across all the arguments made by the Jericho Forum was this common thread, of projecting the experiences of global corporations as prescient for the future reality of business in general. This constructed a business-centred view of information security, rather than a technology-centred one, something made clear when the Forum turned its attention to the opportunities presented by de-perimeterisation.

### Image 4: The opportunistic global enterprise

While technological and organizational changes reduced the efficacy of the perimeter, the ability to step out of this security model was also associated with new business opportunities. The Jericho Forum thus set out a vision of the firm that was tactical, agile, and capable of operating in grey zones outside of its strict control, making use of the ubiquity of public internet infrastructure (rather than costly private networks) in uncertain environments around the globe. As John Meakin, founder member, and former CISO of Standard Chartered Bank, BP and Deutsche Bank, put it in 2007:

If you look for an opportunity in Baghdad—and I only use this as an extreme example—you do not want to set-up a full scale banking office with a proprietary network, your own space and so on, if there is the risk you are going to move quickly to get out [of] there, once you have exploited the business opportunity or once the next bomb goes off. (Dumiak, 2007)

With the widespread availability of the internet, information security could begin to represent itself as a field that enables value, making business possible in otherwise highly insecure spaces. If a de-perimeterised security model was adopted, the internet promised a dramatically reduced *speed-to-market*. Simmonds (2004), in an early Black Hat presentation, quoted Sun Tsu: ‘Cleverness has never been associated with long delays.’ Dorey recalls the opportunity and the challenge faced by many technology professionals in the early 2000s:

We have an opportunity here. We are about to start a new business in country X. Guess what, they have got the internet. We can go live tomorrow. Whereas, if we had to pull our own [corporate link] in some country it could be a year away. (Paul Dorey, interview, 2022)

As routine business and collaboration with third parties took place increasingly over open, public, and distributed networks, it was becoming inconceivable for an organization to ‘rely on a security model that says “it is secure because it runs on my stuff” [or] “data only goes on my network because I know my network is secure”’, as Meakin put it in 2008 (Dumiak, 2008). The intuition of the spatial interior as a secured zone was thus breaking down, and this was presented to the Jericho Forum’s audience as a condition of the market, heralding the fall of the walls. De-perimeterisation appealed to market forces governing technology development and market opportunities governing business priorities, economic logics that lent the movement a sense of inevitability and tied the field to the prerogatives of doing business.

### Image 5: Ink-stained cash

If the Jericho Forum asked practitioners to give up on their assumption that they were protecting the interior of the LAN, what exactly was it that they were supposed to be securing instead? At the heart of de-perimeterisation was a new, value-centric articulation of securing, most clearly visible in a key image of ink-stained cash. This image, of cash stained by ink from a booby-trapped cash canister, is first found in the information security context in a 2001 paper by Jon Measham (2001) of the Royal Mail Research Group Security Team entitled ‘Value-less security’. This paper greatly influenced the founders of the Jericho Forum, and it was here that the term ‘de-perimeterised’ was coined. The image of ink-stained cash would be adopted and used in early Jericho Forum presentations, a pivotal analogy for the clarification of what a ‘de-perimeterised’ security model needed to involve.

The use of ink-staining technology to secure banknotes during transit appeared during the 1980s. One of the pioneers was the Axytrans company, set up by Philippe Regnier in France following the death of a driver working for his cash transport business. Faced with the high costs of preventing theft, Regnier reflects, ‘we asked ourselves, “how can we eradicate temptation?”’ (quoted in Moingeon & Lehmann-Ortega, 2010, p. 276). These ‘intelligent banknote neutralization’ mechanisms were not just an ‘add-on’ measure. In their case study of this innovation, Moingeon & Lehmann-Ortega suggest that they fundamentally perturbed the business model for cash transport. Ink staining, which effectively destroyed the cash value of the banknotes, made it possible to reduce other protections (vans did not need to be so heavily armoured, and guards not so heavily armed) and thus to offer a lighter weight service at lower cost.

By the late 1990s, ink-staining technologies had been introduced into ATMs. Measham observed in his research paper that these machines were lighter weight in design and could be positioned more flexibly, opening up better opportunities to reach customers. The lesson he drew, so central to our story here, was that to understand how such measures secure, one must think deeply about the nature of value. The monetary value of the banknotes was the primary motivator to the attacker. The cash is of equivalent value to the bank (while they may take out insurance, they pay premiums proportionate to the risk of theft). However, the value of the cash in any machine is eclipsed by the revenues the bank can generate from transactions on their ATM network, revenues that depend on service availability, on *how many* machines can be provided and the *convenience* of the sites in which they can be installed.

The cash canister image sketched by Measham and used by the Jericho Forum encapsulates a dense core of ideas that imply a powerful shift, a real rupture in the interpretation of the role of the perimeter and its modalization from a security perspective. The technology that secures may not enclose a surface and defend a generic ‘system’, such as an ATM or a computer network, at all. Security measures must be devised in relation to specific assets, which may be cash, data, or services. Such measures do not necessarily protect in a generic sense of denying all unauthorized access, but rather intervene in the distribution of value, just as the cash canister, in destroying the cash, alleviates the need for a bunker-like ATM, and enables the construction of a more extensive and convenient service network. Assuming that adversaries will be able to operate within a corporate network, de-perimeterisation involves a relentless focus on how value is derived for the business and how it is derived for the attacker, implementing targeted measures that amplify the former and undermine the latter.

The perimeter is transformed. No longer a heavy wall bounding a space, it becomes a light film to be carefully arranged—almost sewn—around the individual asset. In a de-perimeterised security model, therefore, perimeters must not be removed, but narrowed, brought closer to the object to be secured, enveloping it until it takes on its shape. This is a practice that the Jericho Forum defined as ‘shrinking perimeters’. As Simmonds reflects, ‘our argument [was that] at the point you shrink it to protecting one thing there is no more perimeter’ (Paul Simmonds, interview, 2022).

Implied here is a vision of information security integrated into business to such a degree that the two are barely separable. Where traditional information security might be imagined as providing generic secure digital spaces, largely agnostic about the business conducted within them, the cash canister is the symbol of an approach to security in which the business’s core assets are themselves constituted in part by security measures, just as the reach of an ATM network is made possible by hidden booby-trapped ink capsules. The security mechanism ceases to have a distinct form and becomes amorphous: Like the paint sprayed on the cash, it takes a form relative to the asset it is protecting, to the point of becoming *indistinguishable* from it. De-perimeterisation, then, was not simply a discourse on failure. It mobilized positive images of enablement and engagement that blurred the distinction between security and business.

## After de-perimeterisation

### Zero Trust: Chewy centres bite again

The influence of the Jericho Forum has been pervasive. It is also important to recognize, however, that there were concurrent events and projects that intersected with the goals they pursued, that at a minimum created conditions in which de-perimeterisation could take hold and that at a maximum consist of independent articulations of equivalent ideas. An important example is the ‘Black Core’ architecture developed by the US Department of Defense. This came out of the long-running project to build a ‘Global Information Grid’ to support the emerging capability of information operations, requiring seamless integration of real-time data across platforms and locations (Ferris, 2004). The Black Core was an architecture designed to secure a network that included highly sensitive mission-critical information and assets, but that could extend across heterogeneous links into hostile territories. At its heart was the principle of ubiquitous end-to-end encryption (Department of Defense CIO, 2007, p. 28). Black Core would require many of the same technologies, principles and ways of thinking that the Jericho Forum promoted, and this military precedent may have been crucial in shaping the context in which de-perimeterisation could take hold in US government contexts. However, by far the most important landmark in the ascent of de-perimeterisation was the encapsulation of its core principles under what would eventually become its primary brand name: Zero Trust.

In 2010, three years before the Jericho Forum formally closed down, John Kindervag (2010a, 2010b), an analyst at Forrester Research, published a pair of papers: ‘No more chewy centers: Introducing the Zero Trust model of information security’ and ‘Build security into your network’s DNA: The Zero Trust network architecture’. For those familiar with the longer history of de-perimeterisation, there was little in these papers that was new, but they were wrapped in an original metaphorical personification that would go on to prove extremely effective for promoting de-perimeterisation as a concept. The innovation was figurative, bringing the diverse techniques and considerations of de-perimeterisation under a simple logic: security measures, and the architectures they comprise, are personified as embodying either trusting or untrusting attitudes. For Kindervag, the old perimeter is problematic for taking a trusting attitude toward all traffic traversing the network interior. In contrast, the access control, identity management, encryption and monitoring technologies that form the core of a Zero Trust architecture are characterized as ‘untrusting’, in the sense that their default position is one of denying access, denying visibility, and applying ubiquitous surveillance. Applications and users in such an architecture need to have the wherewithal to prove themselves on a recurrent, task-by-task basis.

Kindervag (2010a) presents Zero Trust as a ‘new model for information security’ (p. 1). In a footnote, however, he gives credit to the Jericho Forum, stating that ‘Zero Trust builds upon the deperimeter [sic] ideas first socialized by the Jericho Forum’ (2010a, p. 12). This ‘building on’ involves many of the same technologies and considerations, and rhetorically Zero Trust also builds on the emerging historicization of the perimeter as naïve and outdated. In the opening of ‘No more chewy centers’, Kindervag writes:

There’s an old saying in information security: ‘We want our network to be like an M&M, with a hard crunchy outside and a soft chewy center.’ For a generation of information security professionals, this was the motto we grew up with. It was a motto based on trust and the assumption that malicious individuals wouldn’t get past the ‘hard crunchy outside’. (Kindervag 2010a, p. 1)

Kindervag writes from his own experience. But as we saw above, Cheswick (1990) coined the metaphor of the ‘chewy centre’ precisely to highlight the *problem* of the vulnerable interior. It was not something ‘wanted’, and indeed Cheswick specifically highlighted the risk of insiders (p. 6). But if the historiography of Zero Trust exhibits a degree of selective memory, portraying earlier approaches as more naïve than they really were and appropriating critical stances for a later enlightened era, this is not mere contextualization. However selective, the ability to deploy a narrative about the past that stabilizes a historical differential between an ‘old’ and ‘new’ way of thinking and doing security is a hard-won condition for conceptual and technical evolution (Spencer, 2022b, p. 122).

The publication in 2020 of the US National Institute of Standards and Technology (NIST) Zero Trust Architecture definition was a further landmark, creating a globally recognized reference point for regulators and regulated organizations (NIST information security standards are widely used beyond the US). The NIST document likewise recognizes the debt and provides a clue to how Zero Trust took off.

The work of the Jericho Forum in 2004 publicized the idea of de-perimeterization—limiting implicit trust based on network location and the limitations of relying on single, static defenses over a large network segment. … The concepts of de-perimeterization evolved and improved into the larger concept of zero trust, which was later coined by John Kindervag while at Forrester. Zero trust then became the term used to describe various cybersecurity solutions that moved security away from implied trust based on network location and instead focused on evaluating trust on a per-transaction basis. (Rose et al., 2020, p. 2)

The transition is clear here: Zero Trust became ‘the term used to describe … cybersecurity solutions’, referring to the architecture needed. Where Jericho Forum defined de-perimeterisation in relation to the problems with a superseded architecture, Zero Trust became the name for the new technical architecture. And while the Jericho Forum’s antagonist was an outdated mentality among information security professionals, Zero Trust largely displaced that antagonism onto the now untrusted user. The Jericho Forum were at pains to influence technology vendors, but it took this reconceptualization to marshal the market: with Zero Trust, vendors did not just know what companies wanted, they also knew how to sell it.

The implications are numerous, and beyond the scope of this paper. We might observe, however, a general settling back into a technology-defined model of information security. Just as the firewall provided a model of information security as protecting a perimeter, so apparatuses for access control, identity management, encryption, monitoring and surveillance provide a new model of information security as always suspicious, denying by default, always verifying, always watching. In both cases, we might consider security to be subject to a figurative articulation: Associations inherent to the technology, whether those of enclosure or those of mistrust, create powerful heuristics for securing. Seen in this light, de-perimeterisation is not a simple linear process. Rather, the Jericho Forum created an interlude in which the nature of securing could be questioned, and multiple images and metaphors were deployed to destabilise conventional imagery and provoke new ways of sensemaking. The images of cash canisters, of walls falling, of porous membranes, and so on, introduced contingency into the semantic repertoire of security. What security means was, for a time, up for grabs, and a matter to be questioned. But as de-perimeterisation took hold in the guise of Zero Trust, much of this contingency was lost, as the new technical articulation stabilised itself under a new overarching figuration: securing, like the technologies used for authentication and monitoring, meant adopting a general attitude of mistrust. ‘If individuals know that security is monitoring their actions,’ wrote Kindervag (2010a), using this semantic flexibility of ‘security’, ‘they will be less tempted to do things that are questionable’ (p. 9).

In May 2021, US President Joe Biden issued an executive order, ‘Improving the nation’s cybersecurity’, which, among other things, gave the heads of all US federal agencies 60 days to ‘develop a plan to implement Zero Trust architecture’ (Biden, 2021). These new semantic resources for making sense of security thus found their place as a widespread and increasingly default logic through which the security of systems is justified.

## Discussion: The narrativity of security

An inquiry into a semantic repertoire should not just pick out individual semiotic elements, but should indicate how they hold together as a coherent way of making sense. Likewise, as security logics change, it should be possible to analyse the nature of that change. For this, we turn to the tools of the Greimasian theory of narrativity. In the Greimasian approach, narrative structure implies a core set of actantial roles and relations, including a subject whose project it is; an object of value that is lacking; a need or desire; the source of that need or desire; an anti-subject against which the realization of the object of value must take place; modal objects that make the project possible and are sought in subsidiary projects; and some way in which success or failure is recognized (Greimas, 1987; see also Cooren, 2000, ch.3). At the level of narrative grammar, one may identify securing with any endeavour oriented towards the preservation of some object of value, and associated with the frustration of the goals of an anti-subject. But a Greimasian approach also means that when we talk about security logics, we must account for the figurative level as well, the question of what kind of figures occupy which roles in the schema and what kind of semiotic baggage they bring with them. For instance, logics of national security are traditionally understood by scholars of security in terms of ‘the protection of the boundaries and integrity of the state and its values against the dangers of a hostile international environment’ (Doty, 1998, p. 73). The state, in such a schema, occupies the role of the object of value whose ongoing existence and vitality must be secured against the anti-subject of rival states or terrorist organizations. But in addition to its narrative structure, security is entangled with the complex semiotic potential of the state, and thus with sovereignty, rule, and responsibility, such figurative dimensions conditioning how such a schema can change and be contested.

For information security, de-perimeterisation challenges what kind of actor or entity occupies the role of subject (doing the securing), object of value (being secured), and modal object (making securing possible). The firewall, and the perimeter it guards, had been created as a means to an end, a modal object enrolled in a project of securing a computer network (Cooren, 2000, p. 69). However, through a process of *agencement* (Muniesa et al., 2007), the perimeter came to take the place of the subject, so it is seemingly the perimeter that does the securing, while the information security professional is repositioned into a subservient role as the perimeter’s enabler, making its ongoing operation possible through configuration maintenance and updates. Furthermore, the object of value is overdetermined via the figurative spatial logic of the perimeter, as an interior, a safe space circumscribed by the boundary, secured against a threatening outside. This schema, which suppresses the agential role of the security practitioner and gives the object a spatial determination, is extremely stable, because it draws on the deep cultural repertoire of physical security, of walls, fences, and fortifications.

Cooren suggests that a black box can be understood as ‘a process that is not only “taken for granted”, but also submitted to a narrative schema’. In other words, a black-boxed ‘sub’ mission is encapsulated within the schema of an overarching project (or mission). Indeed, for Cooren (2000), ‘[t]his idea of “submission” … constitutes precisely … the organizing dimension of communication’ (p. 191). The perimeter can be understood as a black box in the sense that it became so well accepted as an element of information security that the assumptions undergirding its status were taken for granted. However, as we have suggested, the control the perimeter exerts is not simply submitted to the task of securing. Its *agencement* effectively inverts the order of mission and sub-mission. Rather than being simply a tool for securing, the perimeter’s own figurative logic, the spatial relations it evokes, come to define the task of securing.

It is in this sense that we take seriously the understanding of their task that the Jericho Forum espoused. A new security model would require that this *agencement* be upset, and the perimeter returned to the position of submission, as a modal object (indeed as just one security measure among many). Once security is no longer submitted to the schema of protecting an interior domain the question of *what needs to be secured* can be opened up, and formulations of information security as constitutive of business value developed.

This shift in the semantic repertoire is not achieved in any single decisive move, but through an aggregation of associations. The trope of Jericho’s falling walls questions the faith of those enclosed in the adequacy of their protection, while sieves and porous membranes challenge the very notion of protection implied by the boundary. The market catalyst draws attention to the conditions that sustain the dominance of perimeter-based architectures (it is not their efficacy that leads to dominance, but the lack of understanding of customers’ real needs by product developers). Tropes of agile exploitation of global business opportunities disturb the association of value with the interior, locating value outside in ‘grey’ zones and insecure spaces. Ink-stained cash challenges simplistic notions of value: The destruction of the cash only makes sense if one understands it in the context of the wider business model, and if one consequently separates the value for the thief from that for the bank. Ultimately, the new relevance of asset value represents an integration of the project of securing into the value-orientation of business, such that the two become entwined.

The rise of Zero Trust represents clear evidence of the success of the Jericho Forum in displacing the perimeter-based model. However, by emphasizing a new technical solution, Zero Trust arguably falls back on a fresh *agencement*, this time not of the perimeter, but of monitoring, identity, access, and encryption technologies. If such an apparatus comes to define what securing means, securing is figuratively recast as a stance of comprehensive mistrust towards all agents and interactions, and the security practitioner is again merely a helper, working to maintain and extend the reach of technologies of mistrust. Such a security logic may help organizations navigate a post-perimeter world, but there remain important tensions likely to undermine ongoing stability and provoke further challenges in the future. If security is understood as an apparatus of mistrust, relationships between security teams and staff are inevitably cast in adversarial terms. This creates an uneasy coexistence between Zero Trust and ideas from safety science that are also gaining influence in information security, ideas suggesting that fostering reliability and resilience depends on effective suppression of tendencies to blame individuals for error (Dekker, 2012).

## Conclusion

Focusing analysis on the semantics of security helps to advance our understanding of information security beyond its technical foundations, and opens up intersections with material semiotics, narrativity, organization theory, and the political economy of technoscience (Birch, 2013). As much as de-perimeterisation is a response to the technical problem of securing interconnected networks, it was achieved through the mobilization of a miscellany of images. It entailed the amplification of a particular set of experiences, especially those of large Western multinational companies, in the new semantic repertoire. It also reoriented security around a concept of asset value (Birch & Muniesa, 2020), and diagnosed the problem and opportunity in relation to markets: markets for security products failing to serve their customers, and market opportunities available to a global enterprise able to reach beyond its home perimeter. Far from being an extrinsic ‘context’ in which information security operates, then, political economy is a medium through which security logics are constructed and deconstructed. These logics play a fundamental role in shaping digital infrastructures, as they provide the means of justification through which designs are assessed, releases approved, products procured, strategies assessed, and incidents analysed. Understanding the narrativity of security, unpacking its logics, is a vital task in understanding the past, present, and future of digital society.

## References

[bibr1-03063127231221107] Baldwin D. A. (1997). The concept of security. Review of International Studies, 23(1), 5–26. 10.1017/S0260210597000053

[bibr2-03063127231221107] Balzacq T. (2005). The three faces of securitization: Political agency, audience and context. European Journal of International Relations, 11(2), 171–201.

[bibr3-03063127231221107] Biden J. R. (2021). Executive order 14028 of May 12, 2021. Improving the nation’s cybersecurity. Federal Register, 86(93), 26633–26647. https://www.govinfo.gov/content/pkg/FR-2021-05-17/pdf/2021-10460.pdf

[bibr4-03063127231221107] Birch K. (2013). The political economy of technoscience: An emerging research agenda. Spontaneous Generations: A Journal for the History and Philosophy of Science, 7(1), 49–61. 10.4245/sponge.v7i1.19556

[bibr5-03063127231221107] Birch K. Muniesa F. (Eds.). (2020). Assetization: Turning things into assets in technoscientific capitalism. MIT Press.

[bibr6-03063127231221107] Bleech N. (2005) De-perimeterisation and the Jericho Forum viewpoint. Open Group. https://collaboration.opengroup.org/jericho/documents/8381/JerichoPres_nb050801.pdf

[bibr7-03063127231221107] Braden R. Clark D. Crocker S. Huitema C. (1994, June). Report of IAB workshop on security in the internet architecture - February 8-10, 1994 (Report No. RFC 1636). Internet Architecture Board. 10.17487/RFC1636

[bibr8-03063127231221107] Buzan B. Wæver O. Wæver O. De Wilde J. (1998). Security: A new framework for analysis. Lynne Rienner Publishers.

[bibr9-03063127231221107] Callon M. Latour B. (1981). Unscrewing the big leviathan: How actors macrostructure reality and how sociologists help them do it. In Knorr-Cetina K. Cicourel A. V. (Eds.). Advances in social theory and methodology: Toward an integration of micro-and macro-sociologies (pp. 277–303). Routledge.

[bibr10-03063127231221107] Carlini F. (2002). Divergenze digitali. Conflitti, soggetti e tecnologie della terza internet. Manifestolibri.

[bibr11-03063127231221107] Cheswick W. (1990). The design of a secure internet gateway [Conference session]. Proceedings of Summer Usenix Conference, Anaheim, California.

[bibr12-03063127231221107] Cheswick W. Bellovin S. (1994). Firewalls and internet security. Repelling the wily hacker. Addison-Wesley Pub.

[bibr13-03063127231221107] Clark D. D. Partridge C. Braden R. T. Davie B. Floyd S. Jacobson V. Katabi D. Minshall G. Ramakrishnan K. K. Roscoe T. Stoica I. Wroclawski J. Zhang L. (2005). Making the world (of communications) a different place. ACM SIGCOMM Computer Communication Review, 35(2), 91–96 10.1145/1070873.1070887

[bibr14-03063127231221107] Collier S. J. Lakoff A. (2015). Vital systems security: Reflexive biopolitics and the government of emergency. Theory, Culture & Society, 32(2), 19–51. 10.1177/0263276413510050

[bibr15-03063127231221107] Collier S. J. Lakoff A. Rabinow P. (2004). Biosecurity: Towards an anthropology of the contemporary. Anthropology Today, 20(5), 3–7. 10.1111/j.0268-540X.2004.00292.x

[bibr16-03063127231221107] Cooren F. (2000). The organizing property of communication. John Benjamins Publishing.

[bibr17-03063127231221107] Cummings J. (2004, September 27). Security in a world without borders. NetworkWorld. https://www.networkworld.com/article/2325411/security-in-a-world-without-borders.html

[bibr18-03063127231221107] Dekker S. (2012). Just culture: Balancing safety and accountability. Ashgate Publishing, Ltd.

[bibr19-03063127231221107] DeNardis L. (2007). A history of internet security. In De Leeuw K. Bergstra J. (Eds.), The history of information security: A comprehensive handbook (pp. 681–794). Elsevier Science & Technology.

[bibr20-03063127231221107] DeNardis L. (2014). The global war for internet governance. Yale University Press.

[bibr21-03063127231221107] Department of Defense CIO. (2007, June). US Department of defense global information grid architectural vision version 1.0. https://www.acqnotes.com/Attachments/DoD%20GIG%20Architectural%20Vision,%20June%2007.pdf

[bibr22-03063127231221107] Doty R. L. (1998). Immigration and the politics of security. Security Studies, 8(2–3), 71–93. 10.1080/09636419808429375

[bibr23-03063127231221107] Dumiak M. (2007). Stay secure outside a bank’s ‘green zone’. Bank Technology News, 20(10), 21.

[bibr24-03063127231221107] Dumiak M. (2008, February). Breaking down the firewall–on purpose. Bank Technology News, 21(2), 17–21.

[bibr25-03063127231221107] Edwards P. N. (1998). Y2K: Millennial reflections on computers as infrastructure. History and Technology, 15(1–2), 7–29. 10.1080/07341519808581939

[bibr26-03063127231221107] Ermoshina K. Musiani F. (2018). Hiding from Whom? Threat models and in-the-making encryption technologies. Intermédialités, 32, 1–25.

[bibr27-03063127231221107] Ferris J. (2004). Netcentric warfare, C4ISR and information operations: Towards a revolution in military intelligence? Intelligence & National Security, 19(2), 199–225. 10.1080/0268452042000302967

[bibr28-03063127231221107] Foucault M. (2007). Security, territory, population: lectures at the Collège de France, 1977-78. Springer.

[bibr29-03063127231221107] Greimas A. J. (1987). On meaning: Selected writings in semiotic theory ( Perron P. J. Collins F. H. , Trans.). University of Minnesota Press.

[bibr30-03063127231221107] Hagström L. Gustafsson K. (2019). Narrative power: How storytelling shapes East Asian international politics. Cambridge Review of International Affairs, 32(4), 387–406. 10.1080/09557571.2019.1623498

[bibr31-03063127231221107] Helmreich S. (2000). Flexible infections: Computer viruses, human bodies, nation-states, evolutionary capitalism. Science, Technology, & Human Values, 25(4), 472–491. 10.1177/016224390002500404

[bibr32-03063127231221107] Ingham K. Forrest S. (2002). A history and survey of network firewalls (Technical report). University of New Mexico.

[bibr33-03063127231221107] Jericho Forum. (2007a). Jericho forum newsletter – may 2007. Open Group. https://collaboration.opengroup.org/jericho/documents/30178/Newsletters.zip

[bibr34-03063127231221107] Jericho Forum. (2007b). Jericho Forum Commandments. Open Group. https://collaboration.opengroup.org/jericho/commandments_v1.2.pdf

[bibr35-03063127231221107] Kindervag J. (2010a). Build security into your network’s dna: The zero trust network architecture. Forrester Research Inc.

[bibr36-03063127231221107] Kindervag J. (2010b). No more chewy centers: The zero trust model of information security. Forrester Research, Inc.

[bibr37-03063127231221107] MacKenzie D. Pottinger G. (1997). Mathematics, technology, and trust: Formal verification, computer security, and the U.S. military. IEEE Annals of the History of Computing, 19(3), 41–59. 10.1109/85.601735

[bibr38-03063127231221107] Measham J. (2001). Value-less security. Can a relativistic approach to risk assessment lead to an extension of the protect, detect, react paradigm? (Technical report). Consignia/Royal Mail.

[bibr39-03063127231221107] Moingeon B. Lehmann-Ortega L. (2010). Creation and implementation of a new business model: A disarming case study. Management (Paris, France: 1998), 13(4), 266. 10.3917/mana.134.0266

[bibr40-03063127231221107] Monsees L. (2020). Cryptoparties: Empowerment in internet security? Internet Policy Review, 9(4), 1–19.

[bibr41-03063127231221107] Muniesa F. Millo Y. Callon M. (2007). An introduction to market devices. The Sociological Review (Keele), 55(s2), 1–12. 10.1111/j.1467-954X.2007.00727.x

[bibr42-03063127231221107] National Research Council. (1990). Computers at risk: Safe computing in the information age. National Academies Press.

[bibr43-03063127231221107] Rose S. Borchert O. Michell S. Connelly S. (2020). Zero trust architecture. NIST Special Publication, 800-207.

[bibr44-03063127231221107] Saita A. (2004, July 29). Beyond borders: Losing the perimeter to gain better data security. SearchSecurity.com (cached copy). https://web.archive.org/web/20051215062519/http://searchsecurity.techtarget.com/originalContent/0,289142,sid14_gci996078,00.html

[bibr45-03063127231221107] Simmonds P. (2004). De-perimeterisation: Border Security is Obsolete… the security challenge for this decade (Conference presentation). Black Hat. https://www.blackhat.com/presentations/bh-europe-04/bh-eu-04-simmonds.pdf

[bibr46-03063127231221107] Slayton R. (2021). Governing uncertainty or uncertain governance? Information security and the challenge of cutting ties. Science, Technology, & Human Values, 46(1), 81–111. 10.1177/0162243919901159

[bibr47-03063127231221107] Smith G. M. (2005). Into cerberus’ lair: Bringing the idea of security to light. British Journal of Politics & International Relations, 7(4), 485–507. 10.1111/j.1467-856X.2005.00204.x

[bibr48-03063127231221107] Spencer M. (2021). Creative malfunction: Finding fault with Rowhammer. Computational Culture, 8, 1–27.

[bibr49-03063127231221107] Spencer M. (2022a). Characterising assurance: Scepticism and mistrust in cyber security. Journal of Cultural Economy. Advance online publication. 10.1080/17530350.2022.2098515

[bibr50-03063127231221107] Spencer M. (2022b). Engines, puppets, promises: The figurations of configuration management. In Lury C. Viney W. Wark S. (Eds.), Figure: Concept and method (pp. 105–125). Springer Nature Singapore. 10.1007/978-981-19-2476-7_6

[bibr51-03063127231221107] Suchman L. Follis K. Weber J. (2017). Tracking and targeting: Sociotechnologies of (in)security. Science, Technology, & Human Values, 42(6), 983–1002. 10.1177/0162243917731524

[bibr52-03063127231221107] Taylor J. R. Van Every E. J. (1999). The emergent organization: Communication as its site and surface. Routledge.

[bibr53-03063127231221107] Thomas D. (2004, August 12). Business to turn up the heat on suppliers. Computing (cached copy) https://web.archive.org/web/20041023073801/http:/www.computing.co.uk/news/1157271

[bibr54-03063127231221107] Tidwell A. S. D. Smith J. M. (2015). Morals, materials, and technoscience: The energy security imaginary in the United States. Science, Technology, & Human Values, 40(5), 687–711. 10.1177/0162243915577632

[bibr55-03063127231221107] Warner M. (2012). Cybersecurity: A pre-history. Intelligence and National Security, 27(5), 781–799. 10.1080/02684527.2012.708530

[bibr56-03063127231221107] Weick K. E. Sutcliffe K. M. Obstfeld D. (2005). Organizing and the process of sense making. Organization Science (Providence, R.I.), 16(4), 409–421. 10.1287/orsc.1050.0133

[bibr57-03063127231221107] Wibben A. T. R. (2011). Feminist security studies: A narrative approach. Routledge.

[bibr58-03063127231221107] Wolff J. (2016). What we talk about when we talk about cybersecurity: Security in internet governance debates. Internet Policy Review, 5(3), 1–13.

[bibr59-03063127231221107] Woolgar S. Neyland D. (2013). Mundane governance: Ontology and accountability. OUP Oxford.

